# The evolution of antimicrobial peptide resistance in *Pseudomonas aeruginosa* is shaped by strong epistatic interactions

**DOI:** 10.1038/ncomms13002

**Published:** 2016-10-03

**Authors:** Nicholas Jochumsen, Rasmus L. Marvig, Søren Damkiær, Rune Lyngklip Jensen, Wilhelm Paulander, Søren Molin, Lars Jelsbak, Anders Folkesson

**Affiliations:** 1 Department of Systems Biology, Technical University of Denmark, Kongens Lyngby, Denmark; 2 Center for Genomic Medicine, Rigshospitalet, 2100 Copenhagen, Denmark; 3 Department of Veterinary Disease Biology, University of Copenhagen, 1870 Frederiksberg C, Denmark; 4 National Veterinary Institute, Technical University of Denmark, Frederiksberg, Denmark

## Abstract

Colistin is an antimicrobial peptide that has become the only remaining alternative for the treatment of multidrug-resistant Gram-negative bacterial infections, but little is known of how clinical levels of colistin resistance evolve. We use *in vitro* experimental evolution and whole-genome sequencing of colistin-resistant *Pseudomonas aeruginosa* isolates from cystic fibrosis patients to reconstruct the molecular evolutionary pathways open for high-level colistin resistance. We show that the evolution of resistance is a complex, multistep process that requires mutation in at least five independent loci that synergistically create the phenotype. Strong intergenic epistasis limits the number of possible evolutionary pathways to resistance. Mutations in transcriptional regulators are essential for resistance evolution and function as nodes that potentiate further evolution towards higher resistance by functionalizing and increasing the effect of the other mutations. These results add to our understanding of clinical antimicrobial peptide resistance and the prediction of resistance evolution.

## Abstract (web summary)

Colistin is an antibiotic used in the treatment of *Pseudomonas aeruginosa* infections in cystic fibrosis patients. Here, Jochumsen *et al*. reconstruct the pathways for the molecular evolution of colistin resistance in *P. aeruginosa* and show that the number of pathways is highly constrained by interactions among genes.

## Graphical abstract

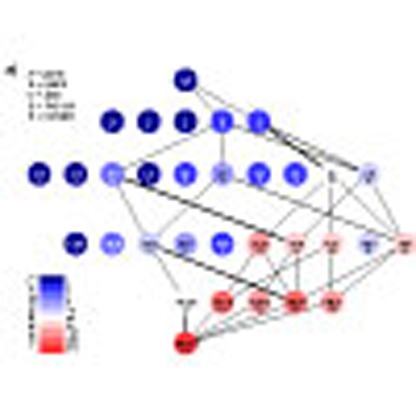

The evolution of antibiotic-resistance has been intensely studied since the first resistant microorganisms were discovered^1^. Theoretical work suggests that clinical use of antibiotics with multiple cellular targets or complex resistance mechanisms could reduce the rate of resistance evolution^2^,^3^. Examples of clinically relevant antibiotics with multicomponent resistance mechanisms are the glycopeptide vancomycin and the cationic antimicrobial peptide colistin^4^,^5^,^6^.

Multicomponent antibiotic resistance is also a suitable model for studying a central biological question: how do adaptations that require acquisition of multiple mutations evolve? The mechanism underlying the evolution of complex resistance mechanisms is not completely elucidated^7^,^8^,^9^,^10^,^11^. Our work aims to shed further light on the evolution of the complex resistance mechanisms that confers high-level colistin resistance in *Pseudomonas aeruginosa*. Colistin is active against many Gram-negative bacteria and is extensively used to treat *P. aeruginosa* lung infections in cystic fibrosis patients^12^,^13^,^14^,^15^. The antimicrobial peptide is one of few remaining effective drugs for treating multidrug-resistant Gram-negative bacteria, in particular *P. aeruginosa*, *Acinetobacter baumannii*, and *Klebsiella pneumonia*^16^. Several mechanisms that confer low-level colistin resistance have been described, but our understanding of the molecular pathways involved in high-level resistance evolution is limited^17^,^18^,^19^,^20^. Among the non-mutational *P. aeruginosa* mechanisms that increase tolerance to antimicrobial peptides is remodelling of the outer membrane by addition of 4-amino-4-deoxy-L-arabinose (L-Ara4N) to the phosphate groups of the lipopolysaccharide (LPS) lipid A region. The enzymes responsible for this modification are encoded by genes in the *arnBCADTEF*-PA3559 (PA3552-PA3559) operon^6^,^21^,^22^,^23^,^24^. Regulation of this operon is complex and involves several distinct two-component regulatory systems that in concert promote *arnB* transcription leading to increased LPS modification in response to low concentrations of divalent ions or the presence of cationic peptides in the environment^17^,^25^,^26^,^27^. *P. aeruginosa* can also acquire low to moderate level resistance through mutations in the two-component regulators *phoPQ* and *pmrAB* depending on strain background and experimental conditions^24^,^28^. However, colistin-resistant clinical isolates from cystic fibrosis patients frequently have high-level resistance (minimum inhibitory concentrations (MICs)>256 μg ml^−1^) indicating that additional factors affect *P. aeruginosa* colistin resistance^19^,^29^,^30^.

We use whole-genome sequencing and experimental evolution to identify the essential components of high-level colistin resistance (col^R^) in *P. aeruginosa*. In addition, we reconstruct evolutionary intermediates to determine the molecular trajectories amenable to evolution of resistance and the influence of epistatic interactions. We show that the evolution of high-level colistin resistance is a complex, multistep process requiring mutations in at least five independent loci that act synergistically to create the phenotype and demonstrating that complex resistance mechanisms can readily evolve *de novo* under the right conditions such as drug gradients or stepwise selection. Moreover, we show that mutations in transcriptional regulators act as evolutionary nodes for further resistance evolution and that the molecular pathways open for resistance evolution are shaped by intergenic epistatic interactions. The identification and understanding of the genetic basis of colistin resistance and the evolutionary process driving resistance evolution may facilitate the development of tools to predict and manage resistance.

## Results

### *pmrB* mutations are insufficient to explain high resistance

**Figure 1:**
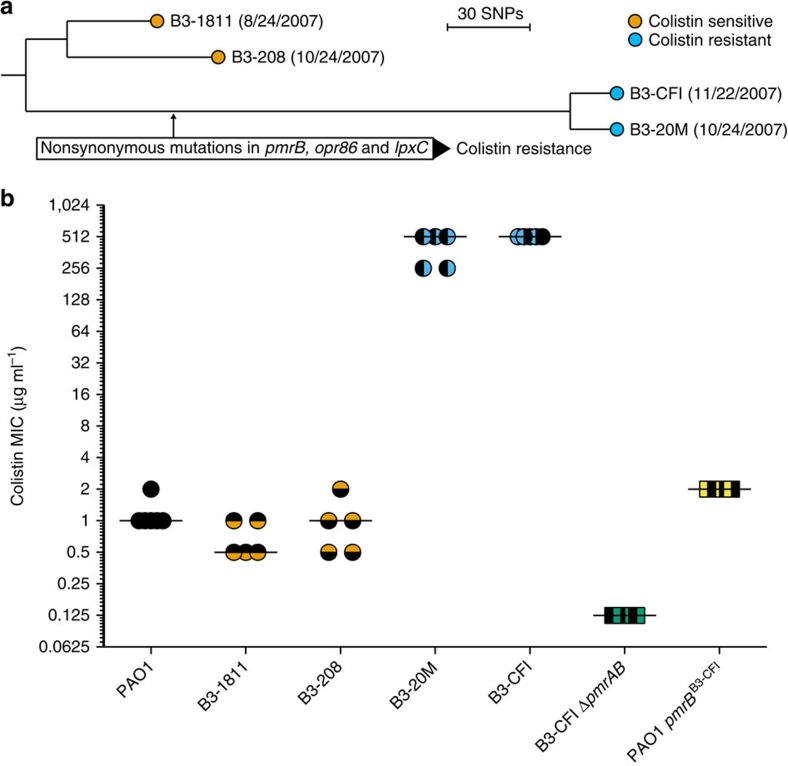
High-level colistin resistance in clinical isolates is multi-factorial. (**a**) Maximum-parsimonious phylogenetic tree representing the evolutionary relationship of *P. aeruginosa* isolates from patient B3. The tree shows the relationship between isolates based on 334 SNPs identified from genome sequencing with an equal number of mutational events (that is, perfect consistency). The *P. aeruginosa* reference isolate PAO1 sequence was used as outgroup to determine the root of the tree. Branch lengths are proportional to number of SNPs between isolates. Date of isolation is in parentheses after the strain designation. (**b**) MICs (μg ml^−1^) from microbroth dilution of *P. aeruginosa* PAO1 (PAO1), the B3 clinical isolates, B3-CFI *pmrAB* deletion (B3-CFI Δ *pmrAB*) and *P. aeruginosa* PAO1 allelic replacement mutant with *pmrB*^634G>A, 742G>A^ allele from B3-CFI (PAO1 *pmrB*^B3−CFI^). Horizontal lines represent median MIC values of three or five replicates. Data underlying the figure is also presented in Supplementary Tables 2 and 3.

We sequenced the genomes of B3-1811, B3-208, B3-20M and B3-CFI, which are four *P. aeruginosa* isolates isolated from consecutive sputum samples from a cystic fibrosis patient with a long-term chronic *P. aeruginosa* lung infection^31^. The patient had undergone three months of oral ciprofloxacin and inhaled colistin therapy^31^. The isolates B3-1811 and B3-208 were colistin sensitive (col^S^) (MIC≤1 μg ml^−1^); and B3-20M and B3-CFI were colistin resistant (col^R^) (MIC>256 μg ml^−1^). We compared the genomes of the four isolates to identify mutations that had accumulated since their most recent common ancestor, that is mutations that most likely accumulated during the three months that the patient underwent antibiotic therapy (Fig. 1). All four isolates contained a 10-bp deletion within *mutL*, which confers a hypermutator phenotype. We also identified 334 single nucleotide polymorphisms (SNPs; 98.8% transitions), and 72 small insertions or deletions (microindels) that differentiated the isolates. The SNPs showed a perfect parsimonious pattern of inheritance (that is the consistency according to a maximum-parsimonious phylogenetic model is 1), reflecting a unidirectional and clonal evolution of the B3 lineage since the most recent common ancestor.

On the basis of the SNPs, we reconstructed the phylogenetic relationship of the isolates (Fig. 1a), and found 198 SNPs and 39 microindels that distinguished the col^R^ isolates from col^S^ isolates (Supplementary Data 1). Two of these mutations were located in *pmrB,* which is involved in regulation of outer membrane LPS modification (Supplementary Table 1, Supplementary Data 1). One of the *pmrB* mutations (742G>A leading to PmrB^A248T^) was previously reported in other clinical col^R^ *P. aeruginosa* isolates and shown to be essential for resistance^19^,^32^. The *pmrAB* genes appeared to be required for high-level resistance, since deletion of *pmrAB* in the col^R^ clinical isolate (B3-CFI) made it more susceptible to colistin (Fig. 1b, Supplementary Table 2). However, introduction of the *pmrB* allele from B3-CFI into laboratory reference strain *P. aeruginosa* PAO1 by allelic replacement produced only a two-fold increase in colistin MIC (Fig. 1b, Supplementary Table 2). Moreover, allelic replacement of *pmrAB* in B3-CFI with the PAO1 allele leads to a decrease in resistance but not to the level of the sensitive B3 strains (Supplementary Table 2). These results suggested that the *pmrB* mutation contributed to the increased colistin resistance but did not fully explain the high-level col^R^ phenotype in the B3-20M and B3-CFI isolates.

### Experimental evolution of colistin resistance

**Figure 2:**
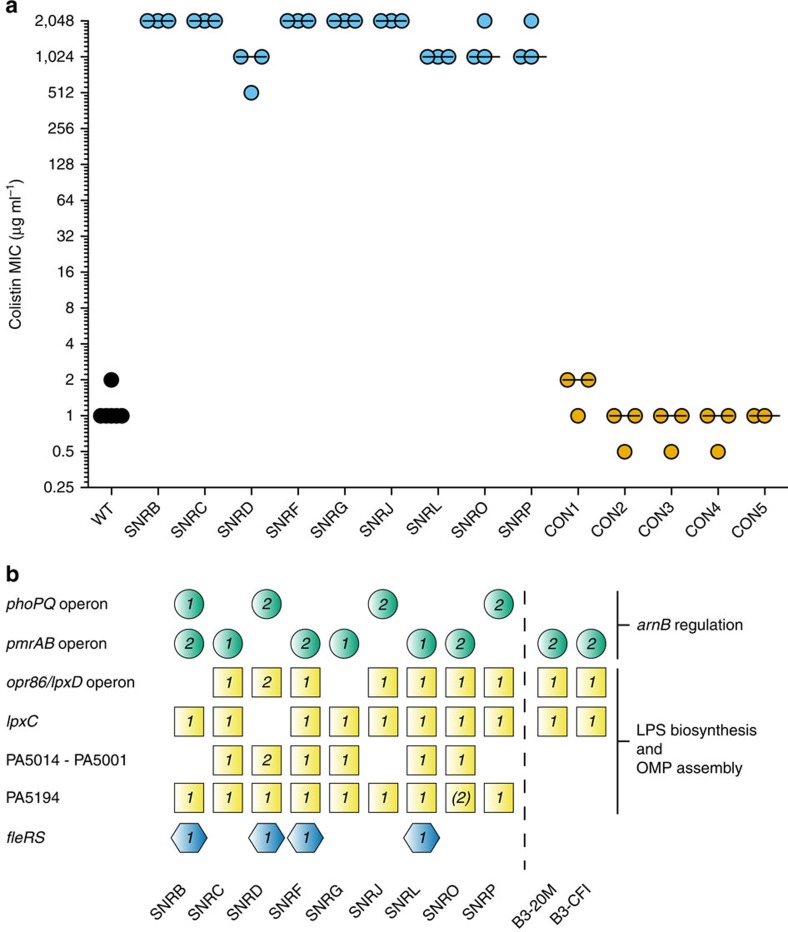
Experimental evolution of high-level colistin resistance in *P. aeruginosa*. (**a**) Colistin MIC values (μg ml^−1^) for Wildtype (WT) ancestral *P. aeruginosa* PAO1; SNRB through SNRP are colistin-resistant clones from surviving selection lineages. CON1 through CON5 are clones from control lineages grown in LB medium in the absence of colistin. CON1 is a mutator strain with mutation in *mutS*. Colistin MIC was determined by microbroth dilution assays. Horizontal lines indicate the median and symbols indicate biological replicates for MIC determination (at least three replicates were used per strain except for CON5). (**b**) Parallel evolution of colistin resistance *in vitro*. Shown are operons or genes with mutations in more than three colistin-resistant strains. The symbols contain the number of mutations at the locus. Strain SNRO did not have a mutation in ORF PA5194 but had a mutation immediately upstream and a mutation downstream of the gene. Circles, genes affecting *arnB*-operon regulation; squares, genes affecting LPS biosynthesis or outer membrane assembly; hexagons, genes affecting other functions; dotted line, *in vitro* selected strains and clinical isolates.

The large number of SNPs separating the col^R^ and col^S^ clinical isolates made it impractical to experimentally establish additional factors underlying high-level resistance in the isolates. We therefore experimentally evolved col^R^ *in vitro* by serial passaging of 16 parallel lineages of *P. aeruginosa* PAO1 with increasing concentrations of colistin for 62 cycles. As controls, five additional lineages were cultured without colistin. Of 16 lineages cultured with colistin, nine developed stable high-level resistance (MICs>256 μg ml^−1^), while seven lineages did not evolve resistance and went extinct during the experiment. All control lineages remained susceptible to colistin (Fig. 2a).

One clone from each of the nine cultures that evolved stable resistance and the five control cultures was selected for whole-genome sequencing. We identified high-quality SNPs and microindels by mapping sequence reads for each strain against the PAO1 reference sequence. Polymorphisms found in all five control strains were excluded from the analysis in order to avoid false-positives caused by strain-specific (native) differences in the parental PAO1 strain used in the experiments relative to the published *P. aeruginosa* PAO1 sequence. All evolved col^R^ strains and one control strain had mutations in the DNA mismatch repair gene *mutS*. Strains under colistin selection accumulated an average of 81 mutations and the control strain (CON1) with the *mutS* mutation had 47 mutations. Only one to three mutations were identified in the genomes of the remaining four control strains (Supplementary Data 1).

To facilitate the identification of mutations related to col^R^, we assumed significant parallel evolution between the nine surviving experimental lineages. We focused on seven independent loci with operons or genes that were nonsynonymously mutated in more than three of the col^R^ strains and with no mutations in control strains (Fig. 2b). Nonsynonymous mutations were identified in *phoP*, *phoQ* and *pmrB* genes, which are known regulators of the LPS modification operon, and in genes affecting lipid A biosynthesis (*lpxC* (PA4406) or *lpxD* (PA3646)). In addition, many strains had mutations in genes encoding components of outer-membrane protein (OMP) assembly (*opr86* (PA3648), seven strains) or core LPS biosynthesis (PA5005, five strains; PA5008, two strains; PA5194, eight strains). In addition, four col^R^ strains had mutations in *fleS/R* (PA1098), which encodes proteins in transcriptional regulation of flagella (Fig. 2b, Supplementary Table 1)^33^. The relative rates of nonsynonymous to synonymous mutations (d*N*/d*S*) of genes mutated in one to two lineages was 0.84 while the ratio in loci mutated in three to nine lineages was 17.3 indicating a strong positive selection of mutations (*P*(*X*≥52)∼pois(*λ*=3)<1 × 10^−15^, where *λ* is the expected number of nonsynonymous SNPs under neutral evolution) (Supplementary Data 1).

### Analysis of evolutionary pathways

**Figure 3:**
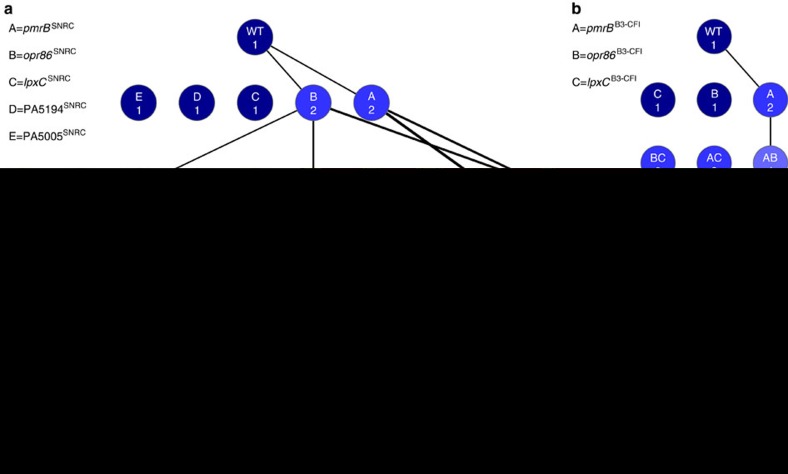
Evolutionary pathways to colistin resistance. (**a**) Phenotypes of the 32 (2^5^) constructed genotypes in PAO1 based on mutations linked to resistance in the colistin-resistant strain SNRC with colistin MIC (μg ml^−1^) for each genotype. (**b**) Phenotypes of the 8 (2^3^) constructed genotypes in PAO1 based on the alleles of *pmrAB*^634G>A,742G>A^ (*pmrAB*^B3−CFI^), *opr86*^1525A>G^ (*opr86*^B3−CFI^) and *lpxC*^551A>G^ (*lpxC*^B3−CFI^) linked to resistance in colistin-resistant strains from patient B3 (B3-20M and B3-CFI) with colistin MIC (μg ml^−1^) for each genotype. For both figures, node labels denote genes affected by the mutation. Blue, low colistin MIC; red, high colistin MIC. Line thickness indicates relative MIC increase. Paths to high-level resistance deemed inaccessible to selection because they had no increase in MIC at any step were omitted for clarity. Wildtype (WT), ancestral *P. aeruginosa* PAO1. Colistin MIC was determined by microbroth dilution assays and values are the median of at least three biological replicates.

To investigate the evolutionary pathways involved in high-level colistin resistance, we randomly chose a single col^R^ strain (SNRC) from the evolution experiment for further investigation. The five resistance-associated mutations that this strain shared with at least three other col^R^ strains were transferred into the ancestral PAO1 strain by allelic replacement (Fig. 3, Supplementary Table 3). Alleles were introduced individually and in combinations to produce a set of 32 (2^5^) genotypes representing all possible combinations of the mutant alleles (Fig. 3a). MIC assays on the constructed genotypes showed that all five mutations contributed to col^R^ (Supplementary Table 3). However, the evolutionary pathways to resistance were constrained because mutations in *lpxC*, PA5005 or PA5194 increased resistance only when combined with other mutations (Fig. 3a). Among the 120 (5!) possible paths to col^R^, only 22 resulted in an increase in drug resistance and were therefore considered amenable to selection (Fig. 3a, Table 1). Only mutations in the evolutionary nodes *pmrB* or *opr86* increased drug resistance in all backgrounds and mutations in these genes provided the two possible first steps to a two-fold increase in col^R^ as measured by MIC (Fig. 3a, Table 1). From the *pmrB* genotype, col^R^ progressed by acquisition of mutations in *lpxC* or *opr86*. From the *opr86* mutant, resistance progressed via mutations in *lpxC* or *pmrB*, or a mutation in PA5005 followed by mutations in *lpxC* or *pmrB*. Trajectories containing *pmrB* mutations were considered more likely to be followed because of their greater effects on col^R^ (Table 1). To investigate if the result of the SNRC reconstruction had a general applicability, we constructed a quintuple *phoQ*^779T>G^ *lpxC*^161C>T^ PA5005^1502A>G^ *opr86*^2305C>T^ PA5194^717G>A^ (NJ268) mutant based on the *phoQ*^779T>G^ allele identified in strain SNRD and a quintuple mutant based on the *phoP*^245G>A^ allele of SNRB by introducing it into strain NJ371 containing the opr86^2305C>T^, *lpxC*^161C>T^, PA5194^717G>A^, PA5005^1502A>G^ mutations from SNRC. The resulting strains exhibited high colistin resistance with MICs of 512 μg ml^−1^ and 1024 μg ml^−1^, respectively, indicating that the effect of the mutations on the resistance phenotype is not restricted to the *pmrB* allele of SNRC strain (Supplementary Table 3). Mutations were also found in *pmrB*, *opr86* and *lpxC* in the col^R^ clinical strains B3-20M and B3-CFI. Introduction of combinations of these mutations into *P. aeruginosa* PAO1 conferred resistance, although *opr86* did not have an effect on its own and *lpxC* increased drug resistance only in *opr86* backgrounds (Fig. 3b and Supplementary Table 2). Moreover, replacing the mutated alleles of *pmrB* and *lpxC* with corresponding alleles from PAO1 in CFI significantly reduced the resistance but the resulting strains were still 32 and 128 times more resistant compared to the sensitive clinical isolates (Supplementary Table 2).

**Figure 4:**
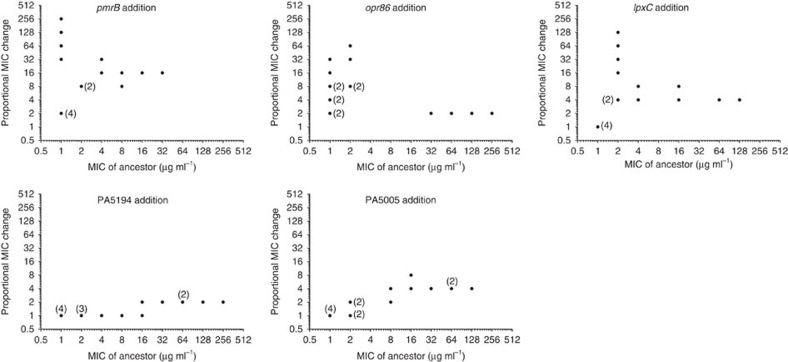
Epistasis in colistin resistance evolution. Graphs show proportional MIC change in 16 allelic backgrounds with increasing MIC upon addition of mutations in *pmrB*, *opr86*, *lpxC*, PA5194 or PA5005 from the colistin-resistant strain SNRC. Each point represents a single strain. In some cases, points are on top of each other as indicated by the numbers in parentheses. Colistin MIC was determined by microbroth dilution assay and presented as the median of at least three biological replicates.

Epistatic interactions have an important influence on resistance evolution^9^. We analysed epistasis among the mutations important for col^R^ by comparing changes in resistance when mutations were added to backgrounds with different fitness, measured as MIC (Fig. 4). The combined effect of the five mutations (512) is 128 times greater than the expected effect of the individual mutations (2 × 2 × 1 × 1 × 1=4) indicating that there is a strong functional relationship between them. The *pmrB*, *opr86* and *lpxC* mutations had the largest effect on resistance in backgrounds with few additional mutations. PA5194 and PA5005 affected resistance only in backgrounds that already had increased resistance (Fig. 4).

### Mutated regulators potentiate high-level resistance evolution

Introduction of mutations in *pmrB*, *phoP*, *phoQ* or *opr86* into *P. aeruginosa* PAO1 resulted in a two-fold increase in MIC, whereas mutations in other genes identified in SNRC did not confer detectable increases in drug resistance (Fig. 3a,b, and Supplementary Table 3). We therefore hypothesized that mutations in key regulators of the LPS modification operon, such as *phoQ* or *pmrB* might constitute critical first steps in the evolution of col^R^. To address this, we conducted an additional serial transfer experiment with *P. aeruginosa* PAO1. In this experiment, 11 of 15 selected lineages were eventually dominated by clones with a 779 T>G mutation causing a V260G shift in PhoQ. In accordance with the hypothesis, we observed that clones carrying this mutation dominated 8 of 11 populations by transfer 13, which corresponded to 6 serial passages at 0.5 μg ml^−1^ colistin and two serial passages at 1 μg ml^−1^ colistin after 5 initial transfers without colistin (Supplementary Fig. 1, Supplementary Table 4).

**Figure 5:**
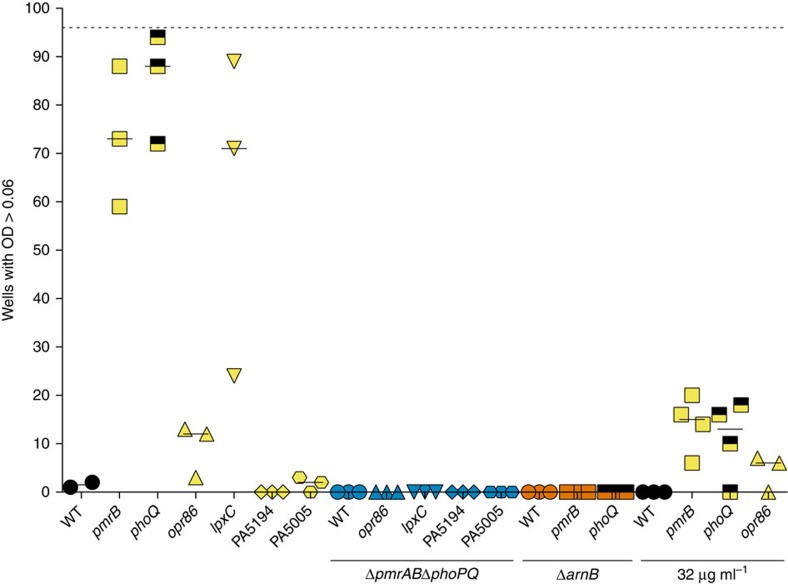
Mutations in regulators potentiate evolution of high-level colistin resistance. Relative estimates for frequency of resistance to 16 μg ml^−1^ colistin (unless otherwise indicated) was determined for different strains of *P. aeruginosa* PAO1 using 96-well microtiter plate assays. Plates were scored for growth after 48 hrs by measuring optical density (OD) at 600 nm. Resistance as growth was scored for wells with OD>0.06. Dotted horizontal line, number of wells (96) investigated; wildtype (WT) *P. aeruginosa* PAO1; *pmrB*, *phoQ*, *opr86*, *lpxC*, PA5194 and PA5005, *P. aeruginosa* allelic replacement mutants with mutations from SNRC or SNRD (*phoQ*^779T>G^). Δ*pmrAB*Δ*phoPQ*, genes encoding the two-component systems were deleted; Δ*arnB arnB* gene of the LPS modification operon was deleted. Each point represents a single experiment. In some cases more than one symbol occupies the same space. Horizontal lines show the median for each strain. Results are based on at least three biological replicates.

To explore the significance of initial mutations on the evolution of col^R^, we used a microtiter resistance assay to investigate the frequency of resistance to 16 μg ml^−1^ and 32 μg ml^−1^ colistin in the reconstructed genotypes. Mutations in *pmrB* and *phoQ* potentiated the evolution of col^R^ because >50% of wells containing strains with these mutant backgrounds showed growth under selection, compared with 0–3% of wells containing strains with wild type, PA5005 or PA5194 backgrounds (Fig. 5). An increased frequency of col^R^ was also observed in *opr86* and *lpxC* mutant backgrounds. However, this increased frequency was abolished in a Δ*pmrAB* Δ*phoPQ* background indicating that the higher frequency was due to mutations in *pmrAB* or *phoPQ.* When the higher colistin concentration was used mutation frequency was reduced to 6–20% and 10–18% for the *pmrB* and *phoQ* mutant, respectively, and 0–7% for the *opr86* mutant (Fig. 5).

These results were consistent with the low resistance of strains lacking mutations in *pmrB* (Fig. 3a). In addition, resistance to colistin required a functional *arnB* operon, supporting the importance of mutations in *pmrB*, *phoP* or *phoQ*, which all increased *arnB* operon expression (Fig. 5, Supplementary Fig. 2).

## Discussion

The molecular mechanisms underlying resistance and tolerance towards antimicrobial peptides in Gram-negative bacteria are very complex where multiple regulators influence susceptibility primarily through a variety of lipopolysaccharide modifications^17^. In this study, we investigated the evolution of colistin resistance in *P. aeruginosa*, a major opportunistic and nosocomial pathogen.

Antibiotic resistance evolution can be severely constrained by intramolecular pleiotropic effects^8^,^9^,^11^,^34^. In these studies, most pathways to higher fitness contained selectively neutral or deleterious steps that made certain trajectories less likely to occur. We showed that this also applies to stepwise evolution of complex antibiotic resistance traits in which multiple genes interact to establish the phenotype. All five mutations that were associated with resistance in SNRC contributed to the resistance phenotype but not in all combinations. Of the five single mutations, only two (*pmrB* and *opr86*) measurably increased resistance alone and these were the only mutations that increased MIC in all backgrounds (Fig. 3a, Table 1). Pathways containing *pmrB* mutations were more likely to lead to high resistance because these mutations had greater effects than *opr86* on col^R^, presumably because of the strong direct effect of the *pmrB* mutation on *arnB* expression (Supplementary Fig. 2).

Mutations in genes encoding regulators are important in the adaptation process since mutations in regulatory elements can affect a broad range of targets, increasing the probability of either beneficial or harmful effects compared to mutations in structural genes^35^,^36^. The evolution of novel traits has recently been addressed within the *Escherichia coli* long-term evolution experiment and is proposed to be a three-step process in which a potentiating background must evolve before a mutation can emerge in the population that actualizes the novel phenotype^37^. We were able to identify molecular details of evolutionary pathways, and we could detect a clear pattern in the process of evolution to high col^R^. At the start of the process, available pathways were highly restricted: specific mutations were required in genes encoding *arnB* operon regulators before additional mutations could express their effects (Fig. 3) and be part of the potentiating process described in the long-term evolution experiment^37^. The PhoPQ and PmrAB two–component systems have previously been implicated in the high colistin resistance phenotype^18^,^19^,^20^. The *parRS* and *cprRS* two-component regulatory systems are additional regulators involved in resistance and tolerance regulation^18^,^20^,^25^,^26^. However, mutations in *parRS* and *cprRS* were not found in our sequence data, indicating that these pathways were not realized in our experiment and in this particular CF patient. Recently, the *cprA* gene has been implicated in affecting polymyxin B resistance^20^. This gene is mutated in the colistin sensitive B3-1811 clinical isolate but not in the highly colistin resistant isolates B3-20M and B3-CFI^31^. It is possible that the genetic variation at the *cprA* locus can explain some of the phenotypic differences in absolute resistance values between the reconstructed strains and the clinical isolates in addition to the variation caused by the multiple mutations due to the mutator phenotype.

In a recent study of the acquisition and reversion of colistin resistance in *P. aeruginosa lpxC* was identified as a key component of resistance evolution *in vitro* in addition to *phoQ* and *pmrB*^38^. The study also shows that colistin resistance is dependent on multiple mutations. However, in that study the mutations quickly reverted to wild type when the strains were grown without selection, a phenomenon not observed in our study^38^.

Clearly, multiple pathways could potentially lead to high colistin resistance. However, only a limited number of these pathways were realized in our study. Recently, Lind *et. al.* 36 have investigated the basis of evolutionary parallelism by removing all of the known pathways that could be mutated to produce the ‘wrinkly spreader’ phenotype in *Pseudomonas fluorescens* and experimentally selected for the phenotype^36^. They showed a large diversity of hidden pathways that had not been discovered earlier^36^ and propose a system of hierarchical rules where mutations in negative regulators have a greater capacity to translate mutation into phenotype due to the larger target size available. If we apply these principles to explain the result of the high colistin resistance evolutionary experiment, we find that regulatory mutations in *pmrB* and *phoQ* potentiated the effect of the mutations in *opr86*, *lpxC*, PA5005 and PA5194. PhoQ acts as a negative regulator of *arnB* expression and *phoQ* was the first gene to get a mutation in the replay experiment, which is consistent with the hierarchal model (Supplementary Fig. 1, Supplementary Table 4). However, in the experiment a specific *phoQ* (PhoQ^V260G^) mutation was preferentially selected, even though any PhoQ-inactivating mutation leads to increased *arnB* operon expression (Supplementary Fig. 2)^28^,^39^,^40^. This specific *phoQ* mutation has also been identified in clinical and *in vitro* evolved isolates with increased col^R^ (refs 30, 38, 41). These findings argue against inactivation of a negative regulator as an adequate explanation for the selective preference of this specific mutation. Moreover, the effects of the *pmrB* mutations are also more complex to be straightforwardly explained as loss of function mutations. The deacetylase *lpxC* and *opr86* are essential genes and affect lipid A biosynthesis and outer membrane (OM) biogenesis^42^,^43^, which suggests that the mutations in these genes are change of function mutations that affect peptide target availability. In Lind *et. al.*^36^ such intragenic activating mutations are one of the least likely category of adaptive mutations making a straight forward translation of the results of the ‘wrinkly spreader’ experiment to high colistin resistance evolution difficult^36^. Further studies are required to reveal the exact function of LpxC and Opr86 mutations and their effect on lipid A biosynthesis^36^. *pmrB* and *opr86* are instrumental in the resistance evolution process. Interestingly, these two genes are among the genes most frequently associated with host adaptation in *P. aeruginosa* CF isolates^44^,^45^. Recently, it has been shown that *pmrB* mutation may be important in the adaptation process to persistent lung colonization. A *pmrB* mutant had significantly increased ability to persist in a chronic respiratory infection mouse model compared with not only the ancestral isolate but also strains adapted to the mouse lung but lacking the mutation^46^. The *pmrB* mutations were selected in the infection model without any use of antimicrobial peptides. It is therefore possible the chronic lung adaptation process selects for mutations that increase the probability of colistin resistance evolution.

Several studies have addressed epistatic interactions on a genomic level^47^,^48^,^49^,^50^. Key discoveries of these studies are that the relative selective benefit of a mutation is influenced by the fitness of the genetic background. The fitness gain from many beneficial mutations is lower in relatively fit backgrounds compared to less fit backgrounds and causing the rate of evolution to decrease over time. The mutations identified here show strong synergistic effects on colistin resistance since the MIC of the *pmrB opr86 lpxC* PA5194 PA5005 mutant was 512 μg ml^−1^, a value 128 fold greater than expected from the individual mutations (2 × 2 × 1 × 1 × 1=4) (Fig. 3a). The mutations in PA5194 and PA5005 were beneficial only in fitter backgrounds (that is, with higher colistin MIC). This observation could be due to that many epistasis studies use growth rate as measure of fitness^48^,^49^. However, biochemical and biophysical constraints mean that growth rate cannot increase indefinitely, which could partly explain the observed diminishing returns of beneficial mutations^48^. In our study, survival of the antibiotic challenge was a primary selective parameter, with growth as a secondary factor once survival was achieved. Therefore, the effect of epistasis on the evolutionary process and the pathways to fitness will depend on the selected trait and the nature of the selection pressure. The evolution of high levels of colistin resistance in *P. aeruginosa* enables the study of the process of adaptation in the context of the clinically relevant area of antibiotic resistance. We show that mutations in key regulators can act as evolutionary nodes for rapid evolutionary change where multiple genetic loci interact to produce a novel trait. Complex resistance mechanisms can therefore readily evolve, only requiring an ordered sequence of mutations where potentiating mutations, which could be selected by unrelated factors such as host adaptation, open up for further resistance evolution. Mutations with little or no individual fitness benefit form strong synergistic interactions when functioning together. More in depth knowledge about the evolution of complex antibiotic resistance mechanisms may facilitate new ways to predict antibiotic resistance evolution and novel tools for resistance management.

## Methods

### Strains and growth conditions

The bacterial strains used in this study are listed in Supplementary Table 5. *Escherichia coli* and *P. aeruginosa* strains were routinely grown in Luria-Bertani (LB) broth. For plasmid maintenance in *E. coli*, medium was supplemented with 8 μg ml^−1^ tetracycline (Tc), 6 μg ml^−1^ chloramphenicol or 100 μg ml^−1^ ampicillin (Ap). For marker selection in *P. aeruginosa*, 30 μg ml^−1^ gentamycin (Gm), 40 μg ml^−1^ tetracycline (Tc), or 200 μg ml^−1^ carbenicillin (Cb) were used as appropriate.

### Serial transfer experiments

Sixteen parallel lineages of *P. aeruginosa* PAO1 were serially propagated in LB medium with increasing concentrations of colistin for 62 transfers. Five additional lineages were cultured in the absence of colistin as controls. Cultures were grown in shaking incubators at 37 °C and transferred twice daily by inoculating into 5 ml fresh medium with 50 μl culture for a daily dilution of 1:10,000. All cultures were initially grown in the absence of colistin for five transfers after which, the positive selection lineages were subjected to a sub-MIC concentration of colistin (0.5 μg ml^−1^). Colistin concentrations were doubled when vigorous growth was observed. By the end of the experiment, 9 of 16 lineages cultured in the presence of colistin had developed stable high-level resistance (MIC>256 μg ml^−1^). Seven lineages were extinct and all control lineages remained susceptible to colistin. One clone from each lineage was isolated and stored in 25% glycerol at −80 °C. Stability of the resistance phenotype was tested by serial passage in LB broth without antibiotic for five passages. All strains remained highly colistin resistant.

### MIC determinations

MIC was determined by microbroth dilution assay in 96-well microtiter plates with LB broth containing two-fold serial dilutions of antimicrobial substance incubated for 24 h according to Wiegand *et al*.^51^. The MIC values reported in this study are the median of at least three biological replicates (except for CON5) and we generally saw very good reproducibility, for example 20 out of the 32 reconstructed strains for the 5 selected mutations in SNRC showed no variation among replicates.

### Mutation frequency estimation

A relative estimate for the frequency of resistance to 16 μg ml^−1^ and 32 μg ml^−1^ colistin was determined for *P. aeruginosa* PAO1 strains by a 96-well microtiter plate assay. Tubes with 10 ml LB medium were inoculated from a single colony and incubated with shaking overnight at 37 °C. The next day, 1.0 × 10^7^ colony forming units were added to wells of 96-well microtiter plates with LB medium containing 16 μg ml^−1^ or 32 μg ml^−1^ colistin and incubated at 37 °C with shaking for 48 h. Plates were scored for growth by measuring optical density at 600 nm (OD600). Growth was defined as OD600>0.06.

### *In vitro* DNA manipulation

Plasmid DNA was purified using NucleoSpin Plasmid EasyPure kit (Macherey-Nagel, Düren, Germany) according to the manufacturer’s instructions. Restriction enzymes were purchased from (Thermo Fisher Scientific, Waltham, USA). PCR was performed in a T3 Thermocycler PCR machine from Biometra using Phusion High-Fidelity DNA Polymerase (Thermo Fisher Scientific, Waltham, USA), primers in Supplementary Table 6 and purified genomic DNA as template. Plasmid DNA was treated with modifying enzymes and restriction endonucleases, ligation of DNA fragments with T4 ligase, and electroporation of bacterial strains were performed using standard methods^52^. In short, PCR products were prepared for blunt-end ligations using Fast DNA End Repair kit (Thermo Fisher Scientific, Waltham, USA), ensuring 5′-phosphorylated, blunt-ended DNA products or was prepared for sticky-end ligation, with primer design ensuring restriction sites for SacI and XbaI. PCR products and digested plasmids were purified NucleoSpin Extract II kit (Macherey-Nagel, Düren,Germany) according to the manufacturer’s instructions. Ligations were performed using the Rapid DNA Ligation Kit (Thermo Fisher Scientific, Waltham, USA) according to manufacturer’s instructions. For blunt-end ligations the pNJ1 plasmid was digested with PstI restriction enzyme and blunt-ended and dephosphorylated, to prevent re-ligations. For sticky-end ligations pNJ1 was digested using SacI and XbaI restriction enzymes. DNA was ligated using Rapid DNA Ligation Kit (Thermo Fisher Scientific, Waltham, USA) according manufacturer’s instructions. The resulting plasmids were transformed into electrocompetent *E. coli CC118*λ*pir* using a Gene Pulser (Bio-Rad, Hercules,CA, USA) as recommended by the manufacturer with 100 μl bacterial cells and 10–100 ng DNA.

### Generation of deletion mutants

Gene disruption cassettes were prepared by PCR as described by Choi and Schweizer^53^ and transformed into plasmid pNJ1 (Supplementary Table 7). Briefly, a set of overlapping PCR reactions were set up using the primers presented in Supplementary Table 6 to produce DNA fragments with a gentamycin resistance marker (GM^R^) flanked by gene specific sequences. These fragments were subsequently blunt-end cloned into the plasmid pNJ1. Gene disruption cassette containing plasmids were transferred into *P. aeruginosa* PAO1 or mutants by triparental mating using the helper strain *E. coli* HB101/pRK600. Allelic exchange as indicated by a sucrose-resistant, Gm-resistant, Tc-sensitive phenotype (or Cb-sensitive when pEX18ApGW plasmids were used) was verified by PCR using gene-specific primers. Generation of unmarked deletion mutants was achieved by transforming electro competent *P. aeruginosa* with pFLP2 as described previously^53^. Briefly, 6 ml of an overnight culture of *P. aeruginosa* PAO1 and mutants, grown in LB medium was harvested in four microcentrifuge tubes by centrifugation (1 min, 1,3000*g*) at room temperature. Each pellet was re-suspended in 1 ml fresh 300 mM sucrose and centrifuged. This was repeated two times and the pellets were then combined in a total of 100 μl 300 mM sucrose. 1 microliter of pFLP2 was added to a 2 mm gap width electroporation cuvette and mixed with 100 μl of electrocompetent cells. The electroporation was performed using the settings: 25 μF, 200 Ω and 2.5 kV. After applying a pulse, 1 ml of pre-warmed LB medium was added immediately, and the cells were transferred to glass test tubes and incubated for 1–1.5 h at 37 °C. After phenotypic expression, the cell suspension was diluted 1:1,000 and 1:10,000 in LB, and 50 μl aliquots were plated on LB+Cb200 plates and incubated over night at 37 °C. Transformants were purified for single colonies on LB+Cb200 plates. Sixteen single colonies were tested for antibiotic-susceptibility on LB+Gm30 plates and on a LB+Cb200 plate. Two Gm^s^ Cb^r^ isolates were streaked for single colonies onto a LB+5% sucrose plate and incubated at 37 °C until sucrose-resistant colonies appeared. Sixteen sucrose-resistant colonies were retested on LB+5% sucrose plates and LB+Cb200 plates Then two sucrose-resistant and Cb^s^ colonies were streaked on LB plates without antibiotics and patched onto LB+Gm30 and LB+Cb200 plates to confirm their Gm^s^ Cb^s^ phenotype. Excision of the Gm^r^ marker and the absence of recombination between remaining FRT sites were verified by colony PCR.

### Genetic reconstruction of intermediates

Mutations from col^R^ strains were transferred via allelic replacement constructs (Supplementary Table 7) into *P. aeruginosa* PAO1 and mutants by triparental mating using the helper strain *E. coli* HB101/pRK600. Merodiploids were selected by plating the conjugation mixture on LB agar plates with 40 μg ml^−1^ Tc and followed by incubation until colonies appeared. Colonies were restreaked on selective plates and single colonies were streaked on 5% (w/v) sucrose LB agar plates (without NaCl). Sucrose-resistant and Tc-sensitive colonies were restreaked on sucrose LB agar plates and screened for the mutated allele by PCR with mutation-specific primers or by PCR followed by restriction fragment length polymorphism (PCR/RFLP) analysis with appropriate restriction enzymes. All mutants were verified by Sanger sequencing at LGC Genomics (Germany). All strains were constructed in the order indicated in Fig. 3 with the exception of the BDE combination (NJ463), which was constructed in the following order: D (NJ81) -> DE (NJ363) -> BDE (NJ463) and the BCDEA (AFP86) strain was constructed by introducing the *pmrB*^*SNRC*^ allele (*pmrB*^524C>T(P175L)^) into BCDE (NJ371).

### Gene expression analysis

Expression of the *arnB* operon was investigated using an *arnB* promoter fusion to green fluorescent protein (GFP)^39^. Three independent, exponentially growing cultures were generated and GFP fluorescence of cells was quantified using a FACSCalibur flow cytometer (Becton Dickinson) and normalized to the GFP fluorescence of the SM2467 strain, which constitutively express GFP.

### Genome sequencing and mutation analysis

Genomic DNA was prepared from *P. aeruginosa* isolates using a Wizard Genomic DNA Purification Kit from Promega and fragmented by acoustic shearing on a Covaris E210 with the standard protocol for 300 bp fragments (duty cycle 10%, intensity 4, cycles per burst 200, time 80 s). Fragmented DNA was purified using Qiaquick PCR purification kit from Qiagen, and end-repaired with End-It DNA End-Repair kit from Epicentre. End-repaired DNA was purified with Agencourt AMPure XP beads (1:1 ratio), and dA was added to the 3′ of blunt DNA fragments using Klenow. DNA was purified using AMPure XP beads (ratio 1:1), and barcoded adaptors with a dT-overhang was ligated to DNA fragments using Fast-Link DNA Ligation kit from Epicentre. DNA was purified using AMPure XP beads (ratio 0.9:1) to remove un-ligated adaptors, and DNA libraries was amplified using standard to Illumina primers to facilitate sequencing on an Illumina GAIIx machine generating 75-bp single reads to obtain a minimum of 2,363,822 reads from each of the genomic libraries.

Mutations were identified by aligning reads to the *P. aeruginosa* PAO1 reference genome (Genbank accession no. NC_002516.2) with Novoalign (Novocraft Technologies)^54^, used GATK for realignment around indels^55^, and produced pileups of the read alignments by SAMtools release 0.1.7 (ref. 56).

SNPs were extracted from the read pileup by the following criteria: (1) quality scores (Phred‐scaled probability of sample reads being homozygous reference) of at least 50 (*i.e.*, *P*≤10^−5^), (2) root-mean-square (RMS) mapping qualities of at least 25, (3) minimum of three reads covering the position and (4) only unambiguous SNP calls. Microindels were extracted from the read pileup by the following criteria: (1) quality scores of at least 500, (2) RMS mapping qualities of at least 25 and (3) support from at least one-fifth of the covering reads as previously described in Marvig *et. al*.^44^.

To avoid false-positives caused by strain-specific (native) differences relative to the published genome sequence of *P. aeruginosa* PAO1, we excluded polymorphisms specific to the genetic background. This meant that polymorphisms found in all five control strains (cycled without colistin) were excluded from analysis of the PAO1-derived isolates, and that polymorphisms shared by all four B3 isolates were excluded from analysis of B3 isolates.

### Data availability

Sequence reads from all isolates are deposited in the Short Read Archive under accession numbers ERP002380 (B3 isolates) and ERP002381 (PAO1 derived isolates). The authors declare that the data supporting the findings of this study are available within the article and its supplementary information files. Bacterial strains and vectors are available from the corresponding author upon request.

## Additional information

**How to cite this article**: Jochumsen, N. *et al*. The evolution of antimicrobial peptide resistance in *Pseudomonas aeruginosa* is shaped by strong epistatic interactions. *Nat. Commun.* **7**, 13002 doi: 10.1038/ncomms13002 (2016).

## Supplementary Material

Supplementary Information Supplementary Figures 1-2, Supplementary Tables 1-7, Supplementary References

Supplementary Data 1 Sequence analysis.

Supplementary Data 2 Minimal inhibitory concentration.

## Figures and Tables

**Table 1:** Effects on colistin resistance of mutations from strain SNRC in the 16 possible allelic backgrounds.

Mutation	Number of backgrounds on which mean mutational effect was			Mean proportional MIC increase*
	Positive	Negative	Negligible†	
*pmrB*	16	0	0	13.5
*lpxC*	12	0	4	6.2
*opr86*	16	0	0	5.7
PA5005	10	0	6	2.2
PA5194	6	0	10	1.3

^*^Geometric mean across all 16 alleles.

^†^≥2-fold changes in minimum inhibitory concentration (MIC) were considered significant.
