# Supplementary material for: The evolution of antimicrobial peptide resistance in Pseudomonas aeruginosa is shaped by strong epistatic interactions
Source: Nat Commun. 2016 Oct 3;7:13002. doi: 10.1038/ncomms13002 (PMC5494192; doi:10.1038/ncomms13002)
Supplement: Supplementary Information — Supplementary Figures 1-2, Supplementary Tables 1-7, Supplementary References [file ncomms13002-s1.pdf]

Supplementary Figures

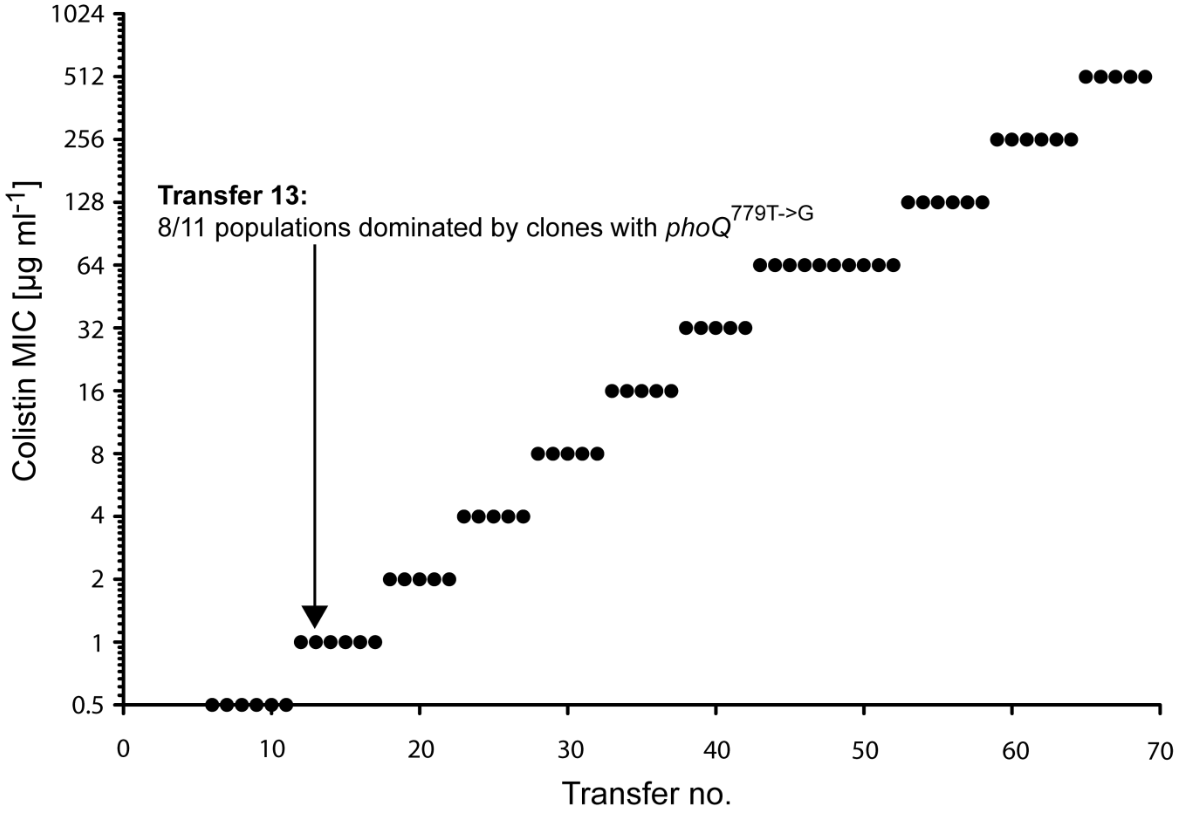

Supplementary Figure 1. Overview of second serial transfer experiments to investigate the potentiating effect of the regulatory mutations. For the first five transfers, no colistin was added to the selected lines. Colistin was added at 0.5 $\mu g\ ml^{-1}$ at transfer six and the concentration was doubled approximately every fifth transfer. Frozen culture samples of the populations were prepared before and after colistin concentration increased. To investigate if mutations in genes such as *phoQ* or *pmrB* constituted a critical first step in colistin resistance evolution, we focused on 11 selected lines. These were eventually dominated by clones with a mutation in *phoQ*$^{779T>G}$. We performed PCR screens for the *phoQ* mutation on a single, randomly chosen clone from each population at transfer 13. This transfer was the earliest point at 1 $\mu g\ ml^{-1}$ colistin for which intermediate populations were available.

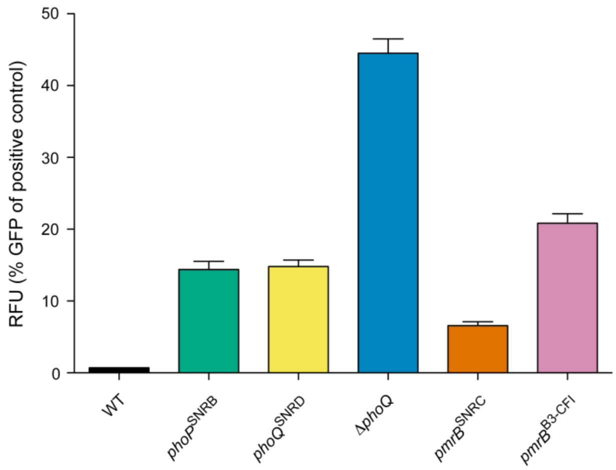

Supplementary Figure 2. Mutations in genes encoding the two-component systems PhoPQ and PmrAB increase *arnB* expression. Expression of *arnB* in Wildtype *P. aeruginosa* PAO1 (WT);, *P. aeruginosa* PAO1 with *phoP*$^{245G>A}$ from SNRB (*phoP*^{SNRB}); *P. aeruginosa* PAO1 with *phoQ*$^{779T>G}$ from SNRD (*phoQ*$^{SNRD)}$; *P. aeruginosa* PAO1 with a deletion in *phoQ* (Δ*phoQ*); *P. aeruginosa* PAO1 with *pmrB* from SNRC (*pmrB*$^{SNRC)}$;, *P. aeruginosa* PAO1 with *pmrB* from B3-CFI (*pmrB*$^{B3\text{-}CFI}$). All strains carried a chromosomally integrated *arnB* promoter fusion to green fluorescent protein (GFP)[1]. Expression of the *arnB* reporter represented as relative fluorescence units (RFU), which is percent fluorescence relative to the positive control strain, SM2467[2]. Values are the average of three independent cultures. Error bars indicate standard deviation.

Supplementary Tables

Supplementary Table 1. Parallel evolution of colistin resistance. Genes and operons with mutations in more than three of the colistin-resistant SNR strains. X = stop codon.

		SNRB	SNRC	SNRD	SNRF	SNRG	SNRJ	SNRL	SNRO	SNRP	B3-20M & B3-CFI
phoPQ	PA1177-*oprH*			1276944 A->G						1276941 T->C	
	oprH-phoP						1277625 C->T				
	phoP	245G>A (G82D)					245G>A (G82D)				
	phoQ			779T>G (V260G)						779T>G (V260G)	
pmrAB	PA4772-PA4773								5361503 T->C		
	PA4773				240G>A (W80_X)						
	PA4774	268T>C (Y90H)									
	PA4775										
	pmrA										
	pmrB	788A>G (Q263R)	524C>T (P175L)		500T>C (L167P)	26T>C (V9A)		154G>A (G52S)	544T>C (W182R)		634G>A (V212M) 742G>A (A248T)
opr86 and *lpxD* operon	*opr86*		2305C>T (P769S)	1598A>G (Q533R) 2295C>T (silent)	1603G>A (D535N)		2305C>T (P769S)	2305C>T (P769S)	2167A>G (T723A)	1964A>G (D655G)	1525A>G (S509G)
	lpxD			707A>G (N236S)							
lpxC	*lpxC*	859G>T (E287_X)	161C>T (A54V)		251C>T (A84V)	251C>T (A84V)	251C>T (A84V)	725C>T (A242V)	251C>T (A84V)	161C>T (A54V)	551A>G (E184G)
PA5014-PA5001	PA5005		1502A>G (H501R)	1502A>G (H501R)	61T>C (Y21H)			1025C>T (P342L)	413A>G (H138R)		
	PA5008			220T>C (W74R)		211A>G (K71E)					
PA5194	PA5194	509T>C (L170P)	717G>A (W239X)	62T>C (L21P)	678G>A (W226X)	33G>A (W11X)	717G>A (W239X)	378G>A (W126X)		676T>C (W226R)	
fleRS	*fleS*	27C>T (silent)						829C>T (Q277X)			
	fleR	1352C>T (T451I)		1325C>T (A442V)	743A>G (E248G)						

Supplementary Table 2. Colistin microbroth dilution MIC values [µg ml^{-1}] for clinical isolates and strains related to these. A = *pmrB*$^{634G>A\ (V212M)\ 742G>A\ (A248T)}$, B = *opr86*$^{1525A>G\ (S509G)}$, C = *lpxC*$^{551A>G\ (E184G)}$.

Strain ID	Ancestor	Mutation combination	MIC	MIC	MIC	MIC	MIC	MIC
B3-1811	-	Clinical isolate	1	0.5	1	0.5	0.5	
B3-208	-	Clinical isolate	1	0.5	2	0.5	1	
B3-20M	-	Clinical isolate	256	512	512	512	256	
B3-CFI	-	Clinical isolate	512	512	512	512	512	
NJ622	B3-CFI	Δ*pmrAB*	0.125	0.125	0.125			
AFP99	B3-CFI	*pmrAB*^{PAO1}	32	64	32			
AFP100	B3-CFI	*lpxC*^{PAO1}	64	128	128			
PAO1	-	-	1	1	1	1	1	2
NJ104	PAO1	A	2	2	2			
NJ385	PAO1	B	1	1	2			
NJ95	PAO1	C	1	1	1			
NJ386	NJ104	AB	4	4	8	8	4	
NJ118	NJ104	AC	2	2	2	2	4	
NJ432	NJ385	BC	2	2	2			
NJ388	NJ386	ABC	32	16	32	32	32	

Supplementary Table 3. Colistin microbroth dilution MIC values [µg ml^{-1}] for laboratory evolved strains and strains related to these. A = $\textit{pmrB}^{\text{524C>T (P175L)}}$, B = $\textit{opr86}^{\text{2305C>T (P769S)}}$, C = $\textit{lpxC}^{\text{161C>T (A54V)}}$, D = $\text{PA5194}^{\text{717G>A (W239stop)}}$ and E = $\text{PA5005}^{\text{1502A>G (H501R)}}$.

Strain ID	Ancestor	Mutation combination	MIC	MIC	MIC	MIC	MIC	MIC
PAO1	-	-	1	1	1	1	1	2
SNRB	PAO1	Evolved	2048	2048	2048			
SNRC	PAO1	Evolved	2048	2048	2048			
SNRD	PAO1	Evolved	1024	512	1024			
SNRF	PAO1	Evolved	2048	2048	2048			
SNRG	PAO1	Evolved	2048	2048	2048			
SNRJ	PAO1	Evolved	2048	2048	2048			
SNRL	PAO1	Evolved	1024	1024	1024			
SNRO	PAO1	Evolved	2048	1024	1024			
SNRP	PAO1	Evolved	1024	2048	1024			
CON1	PAO1	Evolved	2	2	1			
CON2	PAO1	Evolved	1	1	0.5			
CON3	PAO1	Evolved	1	1	0.5			
CON4	PAO1	Evolved	1	1	0.5			
CON5	PAO1	Evolved	1	1				
$\textit{phoQ}^{\textit{SNRD}}$	PAO1	$\textit{phoQ}^{\text{779T>G (V260G)}}$	2	2	2			
NJ580	PAO1	*ΔphoQ*	2	2	1			
NJ582	PAO1	$\textit{phoP}^{\text{245G>A (G82D)}}$	2	2	2			
$\textit{pmrAB}^{\textit{SNRC}}$	PAO1	A ($\textit{pmrB}^{\text{524C>T (P175L)}}$)	2	2	2	2	2	
NJ27	PAO1	B	2	2	2			
NJ49	PAO1	C	1	1	1			
NJ81	PAO1	D	1	1	1			
NJ176	PAO1	E	1	1	1			

NJ30	*pmrAB*^{SNRC}	AB	32	16	16	16	16	
NJ102	*pmrAB*^{SNRC}	AC	32	16	32			
NJ83	*pmrAB*^{SNRC}	AD	2	2	2			
NJ179	*pmrAB*^{SNRC}	AE	2	2	2			
NJ58	NJ27	BC	8	4	8			
NJ88	NJ27	BD	2	2	2			
NJ360	NJ27	BE	4	4	4			
NJ99	NJ49	CD	1	1	1			
NJ362	NJ49	CE	1	1	1			
NJ363	NJ81	DE	1	1	1			
NJ97	NJ30	ABC	64	64	64			
NJ89	NJ30	ABD	32	16	16			
NJ445	NJ30	ABE	64	64	64			
NJ112	NJ102	ACD	64	64	16			
NJ373	NJ102	ACE	128	128	64			
NJ365	NJ83	ADE	2	2	2			
NJ92	NJ58	BCD	8	8	16			
NJ405	NJ58	BCE	16	16	16			
NJ463	NJ363	BDE	4	4	4			
NJ407	NJ99	CDE	1	1	2			
NJ110	NJ97	ABCD	128	128	256			
NJ181	NJ97	ABCE	256	256	256			
NJ370	NJ89	ABDE	128	128	128			
NJ375	NJ112	ACDE	256	256	128			
NJ371	NJ92	BCDE	32	32	16			
NJ182	NJ110	ABCDE	512	512	512			
AFP86	NJ371	BCDEA	512	512	512			

NJ268	NJ161	*phoQ*$^{779T>G\ (V260G)}$ CEBD	512	512	512	256	256		
NJ528	NJ371	BCDE (*phoP*$^{245G>A\ (G82D)}$)	1024	1024	512				
NJ588	NJ371	BCDE (Δ*phoQ*)	1024	512	512				

Supplementary Table 4. PCR screen for *phoQ*$^{779T>G}$ in populations from the second serial transfer experiment investigating the potentiating effect of mutations in regulators. A single, randomly chosen clone from each population at transfer 13 (corresponding to 6 serial passages at 0.5 μg ml^{-1} colistin and 2 passages at 1 μg ml^{-1} colistin) was used for the PCR template. The *phoQ* mutation reached fixation in all populations by the end of the experiment.

	PAO1 WT	NA1	NA3	NA4	NA6	NA7	NA8	NA10	NA11	NA12	NA13	NA15
Mutation present	*No*	*Yes*	*Yes*	*No*	*Yes*	*Yes*	*Yes*	*No*	*No*	*Yes*	*Yes*	*Yes*

Supplementary Table 5. Bacterial strains used in the study.

**Bacterial strains**	**Relevant characteristics**	**Reference**
P. aeruginosa		
PAO1	Wild type	[3]
B3-1811	Colistin-sensitive clinical isolate from patient B3	[4]
B3-208	Colistin-sensitive clinical isolate from patient B3	[4]
B3-20M	Colistin-resistant clinical isolate from patient B3	[4]
B3-CFI	Colistin-resistant clinical isolate from patient B3	[4]
SNRB	Colistin-resistant clone from *in vitro* experiment	This study
SNRC	Colistin-resistant clone from *in vitro* experiment	This study
SNRD	Colistin-resistant clone from *in vitro* experiment	This study
SNRF	Colistin-resistant clone from *in vitro* experiment	This study
SNRG	Colistin-resistant clone from *in vitro* experiment	This study
SNRJ	Colistin-resistant clone from *in vitro* experiment	This study
SNRL	Colistin-resistant clone from *in vitro* experiment	This study
SNRO	Colistin-resistant clone from *in vitro* experiment	This study
SNRP	Colistin-resistant clone from *in vitro* experiment	This study
CON1	Colistin-sensitive clone from *in vitro* experiment	This study
CON2	Colistin-sensitive clone from *in vitro* experiment	This study
CON3	Colistin-sensitive clone from *in vitro* experiment	This study
CON4	Colistin-sensitive clone from *in vitro* experiment	This study
CON5	Colistin-sensitive clone from *in vitro* experiment	This study
SM2467	PAO1 *att*TN7-$P_{A1/04/03}$::*gfp*^{mut3*}, Gm^{r}	[2]
NJ271	PAO1 *ΔpmrABΔphoPQ PA5005*$^{1502A>G}$	This study
NJ272	PAO1 *ΔpmrABΔphoPQ lpxC*$^{161C>T}$	This study
NJ497	PAO1 *ΔpmrABΔphoPQ*	This study
NJ464	PAO1 *ΔpmrABΔphoPQ PA5194* $^{717G>A}$	This study
NJ460	PAO1 *ΔpmrABΔphoPQ opr86*$^{2305C>T}$	This study
NJ510	PAO1 *ΔarnB*	This study
NJ550	PAO1 *pmrAB*$^{52\ C>T}$ *ΔarnB*	This study
NJ551	PAO1 *phoQ*$^{779T>G}$ *ΔarnB*	This study
E. coli		
CC118 λpir	*E. coli* CC118 lysogenized with lambda pir phage	[5]
HB101	*recA* ,thi, pro, leu, hsd RM^{+}; Sm^{R}	[6]

Supplementary Table 6. Primers used in the study.

Primers	Sequence (5' → 3')
Gm-F	CGAATTAGCTTCAAAAGCGCTCTGA
Gm-R	CGAATTGGGGATCTTGAAGTTCCT
GW-*att*B1	GGGGACAAGTTTGTACAAAAAAGCAGGCT
GW-*att*B2	GGGGACCACTTTGTACAAGAAAGCTGGGT
PA3552-UpF-GWL	TACAAAAAAGCAGGCTTCCCCATGTTTCCATGAAGT
PA3552-UpR-Gm	TCAGAGCGCTTTTGAAGCTAATTCGGGAGAATGGCAGAAAGTCCA
PA3552-DnF-Gm	AGGAACTTCAAGATCCCCAATTCGGTAGCGGCATCCATTTCATC
PA3552-DnR-GWR	TACAAGAAAGCTGGGTTAGTTGCGGTTGAGGATCAC
PA4776-F-SphI	GAGAGCATGCCTGATCGTCGCCATCCTCCT
PA4777-R-XbaI	ACATTCTAGACCGACTTCGTCGAGGGAGAA
PhoPhindIII	GAGAAAGCTTCTGAGCAAGAATGCCTCGAT
PhoQxbaI	GAGATCTAGAGGCGAGAAAGAACAGCACAC
opr86_1F	GATGGCGGTAGGATGGATAC
opr86_3R	GCTACAACTTCGGCTTCGTC
lpxC_2F	GCGAACTTAGCGAAAATCCTT
lpxC_3R	CGTCAGGCCGATTGAAGTAA
lpxC_4R	GATCACCATCCCGAACGA
PA5194_2F	CTGGAACACTCCACCATCAA
PA5194_4R	TTGGAGCTGCGAATGCTCTT
phoQ-UpF-SacI	CATAGAGCTCCGCATCCTCGAATACCTCAT
phoQ-UpR	TCAGAGCGCTTTTGAAGCTAATTCGGCGTGTAGTTGATCGACTCGT
phoQ-DnF-2SOE	CGAATTAGCTTCAAAAGCGCTCTGAACCTGCTGGAGAACGCCTAT
phoQ-DnR-XbaI	ACATTCTAGAAGATCGCTTCGCTCAGGTT
PA5005-F	TACTTCTCGCCCAAGCTGAT
PA5005-R	CGGTTGTAGAGCGTCTCGAT
opr86_full_XbaI	GAGATCTAGAGATGGCGGTAGGATGGATAC
opr86_full_SacI	ACATGAGCTCCATCGCCAATTTCCTACTGG
pmrB_UpFSacI	CATAGAGCTCGTCGGCTTCGTGCTGTGC
pmrB_UpRGm	TCAGAGCGCTTTTGAAGCTAATTCGAACGCCGAACCAGACCA
pmrB_DnFGm	AGGAACTTCAAGATCCCCAATTCGGAGCGCATCAGCACCTTG
pmrB_DnRPstI	AGAGACTGCAGGGCAGGAACACCTGCACTTC

Supplementary Table 7. Plasmids used in the study.

Plasmids	Relevant characteristics	Reference
pNJ1	Tc^R *sacB*$^+$ R6K *ori*, mobRP4, Allelic replacement vector derived from pDS132	[7]
pBK-mini-Tn7- P*arnB*::*gfp*	Delivery plasmid for mini-Tn7-P*arnB*::*gfp*; Ap^r Gm^r	[1]
pUX-BF13	*mob*$^+$ *ori*-R6K; helper plasmid providing the Tn7 transposition functions *in trans*; Ap^r	[8]
pPS856	0.83 kb blunt-ended *Sac*I fragment from pUCGM ligated into the *Eco*RV site of pPS854; Ap^r, Gm^r	[9]
pFLP2	2.6 kb *Bam*HI–*Sph*I fragment from pALB2 ligated between the same sites of pPS908; Ap^r, Cb^r	[9]
pRK600	ori-ColE1 RK2-mob$^+$ RK2-tra$^+$, helper plasmid for conjugation; Cm^r	[6]
pEX18ApGW-*phoPQ*::Gm	*phoPQ*::Gm knockout vector; Suc^s Ap^r Gm^r	[1]
pEX18ApGW-*pmrAB*::Gm	*pmrAB*::Gm knockout vector; Suc^s Ap^r Gm^r	[1]
pNJ9	1.979 kb lpxC 2F/4R PCR fragment from PAO1 wt, phosphorylated and ligated into dephos. blunt-ended pNJ1 (cut with PstI)	
pNJ10	Δ*pmrB*::Gm SOE PCR fragment from PAO1/pPS856, phosphorylated and ligated into dephos. blunt-ended pNJ1 (cut with PstI)	This study
pNJ11	1.875 kb opr86 1F/3R PCR fragment from SNRC, phosphorylated and ligated into dephos. blunt-ended pNJ1 (cut with PstI)	This study
pNJ12	1.979 kb lpxC 2F/4R PCR fragment from SNRC, phosphorylated and ligated into dephos. blunt-ended pNJ1 (cut with PstI)	This study
pNJ13	pmrB UpF/DnR fragment from B3-CFI, phosphorylated and ligated into dephos. blunt-ended pNJ1 (cut with PstI)	This study
pNJ14	lpxC 2F/3R fragment from B3-CFI, phosphorylated and ligated into dephos. blunt-ended pNJ1 (cut with PstI)	This study
pNJ15	PA5194 2F/4R fragment from SNRC, phosphorylated and ligated into dephos. blunt-ended pNJ1 (cut with PstI)	This study
pNJ17	PA5005 fragment from SNRC, phosphorylated and ligated into dephos. blunt-ended pNJ1 (cut with PstI)	This study
pNJ23	ΔarnB::Gm PCR fragment phosphorylated and ligated into dephos. blunt-ended pNJ1 (cut with PstI)	This study
pNJ32	opr86_full_xbaI/sacI PCR fragment from B3-CFI, cut with XbaI and SacI and ligated into SacI/XbaI cut pNJ1	This study
pNJ34	phoPHindIII/phoQXba PCR fragment from SNRB, phosphorylated and ligated into dephos. dlunt-ended pNJ1 (cut with PstI)	This study
pNJ36	ΔphoQ 2SOE PCR fragment, cut with SacI XbaI and ligated into SacI XbaI digested pNJ1	This study
pNJ37	pmrB PA4776FSphI/PA4777RXbaI fragment from SNRC, phosphorylated and ligated into dephos. blunt-ended pNJ1 (cut with PstI)	This study
pNJ38	phoPHindIII/phoQXbaI PCR fragment from SNRD, phosphorylated and ligated into dephos. dlunt-ended pNJ1 (cut with PstI)	This study
pNJ49	PA4776FSphI/PA4777RXbaI fragment from PAO1, phosphorylated and ligated into dephos. blunt-ended pNJ1 (cut with PstI)	This study

Supplementary References

1. Jochumsen N, Liu Y, Molin S, Folkesson A. A Mig-14-like protein (PA5003) affects antimicrobial peptide recognition in Pseudomonas aeruginosa. *Microbiology* **157**, 2647-2657 (2011).

2. Klausen M, *et al.* Biofilm formation by Pseudomonas aeruginosa wild type, flagella and type IV pili mutants. *Mol Microbiol* **48**, 1511-1524 (2003).

3. Stover CK, *et al.* Complete genome sequence of *Pseudomonas aeruginosa* PA01, an opportunistic pathogen. *Nature* **406**, 959-964. (2000).

4. Marvig RL, *et al.* Draft Genome Sequences of Pseudomonas aeruginosa B3 Strains Isolated from a Cystic Fibrosis Patient Undergoing Antibiotic Chemotherapy. *Genome Announc* **1**, 00804-00813 (2013).

5. de Lorenzo V, Herrero M, Jakubzik U, Timmis KN. Mini-Tn5 transposon derivatives for insertion mutagenesis, promoter probing, and chromosomal insertion of cloned DNA in gram-negative eubacteria. *J Bacteriol* **172**, 6568-6572 (1990).

6. Kessler B, de Lorenzo V, Timmis KN. A general system to integrate lacZ fusions into the chromosomes of gram-negative eubacteria: regulation of the Pm promoter of the TOL plasmid studied with all controlling elements in monocopy. *Mol Gen Genet* **233**, 293-301 (1992).

7. Yang L, *et al.* Polysaccharides serve as scaffold of biofilms formed by mucoid Pseudomonas aeruginosa. *FEMS Immunol Med Microbiol* **65**, 366-376 (2012).

8. Bao Y, Lies DP, Fu H, Roberts GP. An improved Tn7-based system for the single-copy insertion of cloned genes into chromosomes of gram-negative bacteria. *Gene* **109**, 167-168 (1991).

9. Hoang TT, Karkhoff-Schweizer RR, Kutchma AJ, Schweizer HP. A broad-host-range Flp-FRT recombination system for site-specific excision of chromosomally-located DNA sequences: application for isolation of unmarked Pseudomonas aeruginosa mutants. *Gene* **212**, 77-86 (1998).
